# Retraction pattern of delaminated rotator cuff tears: dual-layer rotator cuff repair

**DOI:** 10.1186/s13018-016-0407-9

**Published:** 2016-07-06

**Authors:** Sang-Won Cha, Choon-Key Lee, Hiroyuki Sugaya, Taegyun Kim, Su-Chan Lee

**Affiliations:** Department of Orthopaedic Surgery, Busan Himchan Hospital, 255 Chungnyeol-daero, Dongnae-gu, Busan 47813 Korea; Shoulder and Elbow Center, Funabashi Orthopaedic Hospital, 1-833 Hazama, Funabashi, Chiba 2740822 Japan; Department of Orthopaedic Surgery, Mokdong Himchan Hospital, 120 Shinmok-ro, Yangcheon-gu, Seoul 07999 Korea

**Keywords:** Shoulder, Rotator cuff tear, Delaminated, Delamination, Tear pattern

## Abstract

**Background:**

There has been no report to date regarding retraction patterns of delaminated rotator cuff tears. The purpose of this study was to evaluate the incidence and tearing patterns of delamination and repair integrity after the dual-layer repair of delaminated cuff tears.

**Methods/design:**

A consecutive series of 64 patients with posterosuperior rotator cuff tears underwent arthroscopic rotator cuff repair from August 2011 to September 2012. Among the patients, 53 who received either dual-layer double-row (DLDR) repair or dual-layer suture bridge (DLSB) repair and 11 who received a single-layer repair were evaluated. The mean follow-up period after surgery was 26.5 months. The retraction direction of the deep and superficial layers was analyzed, and the integrity of the repaired constructs was determined in 37 patients through magnetic resonance imaging at a mean of 12.1 months after surgery.

**Results:**

The incidence of delamination was 82.8 %. Posteromedial retraction of the deep and superficial layers was observed in 98.1 and 88.5 % of cases, respectively. The Constant score and UCLA score increased preoperatively to postoperatively, showing no significant differences between the dual-layer repair group and single-layer repair group. Retear was found in two (7.6 %) patients in the dual-layer repair group and three (27.2 %) patients in the single-layer repair group (*p* = 0.016).

**Conclusions:**

Differential rotator cuff repair strategies are needed to address rotator cuff tears, since recent studies have changed our concept of rotator cuff tears. We have focused on three areas: incidence, retraction patterns, and clinical outcomes. The incidence of delamination was extremely high. Both the superficial layer and deep layer were mainly retracted posteromedially. The retraction of the deep layer and superficial layer may be affected by the infraspinatus. Dual-layer rotator cuff repair based on the retraction pattern of delamination is recommended for improvement of repair integrity and of clinical outcomes.

## Background

Delamination is a horizontal, partial thickness split of the tendon substance between layers of a ruptured rotator cuff. A widely ranging incidence of 38–92 % has been reported, but the precise cause of delamination is still unknown [1–5]. Sonnabend et al. [6] demonstrated through a histological study that laminated tears of the rotator cuff generally occurred between two layers of differing collagen fiber orientation. In their anatomic study on the humeral insertion of the rotator cuff, Mochizuki et al. [7] reported that the infraspinatus tendon (ISP) may be involved in a high proportion of rotator cuff tears, since the infraspinatus has a substantially wider footprint on the greater tuberosity than had been previously believed. Understanding of the anatomic footprint and retraction patterns of each layer in delaminated rotator cuff tears based on these histological and anatomic studies may promote a more ideal recovery environment following rotator cuff repair by allowing anatomical balancing and appropriate tensioning of the rotator cuff repair. However, only a few studies [3, 6, 8–10] have described the causes, characteristics, or surgical methods involved in delamination, and none of them has discussed the actual retraction direction of the delaminated cuff, the reduction direction during dual-layer rotator cuff repair, or structural recovery from delamination after repair.

The purpose of our study was to determine the incidence of delamination, evaluate the patterns and characteristics of delamination, and analyze whether the presence of delamination affects the outcome of rotator cuff repairs. The working hypotheses included: (1) the incidence of delamination would be higher than the incidence of 38 to 82 % found in previous reports; (2) the retraction pattern of delamination would be affected by involvement of the infraspinatus; and (3) dual-layer (anatomically balanced) repair based on the pattern of delaminated rotator cuff tears may enhance structural rigidity and improve structural outcomes by avoiding undue tension on repairs.

## Methods/design

### Patient selection

From August 2011 to September 2012, 64 consecutive patients underwent arthroscopic primary rotator cuff repair at our clinic. Rotator cuff tears were confirmed by preoperative MRI in all patients. Inclusion criteria were as follows: full-thickness rotator cuff tears undergoing arthroscopic rotator cuff repair and a minimum follow-up of 1 year. Exclusion criteria were partial thickness tears, isolated subscapularis tears, revision rotator cuff repair, and clinical findings of instability and arthritis. This study protocol was approved by the Hospital Ethnic Committee of Himchan Hospital Health System (no.: 01-2013; date: February 18, 2013), and a signed consent form was obtained from each subject. Preoperative functional assessment was obtained using the UCLA and the Constant score (CS) in all 64 cases. Among them, 53 patients with delaminated posterosuperior rotator cuff tears and 11 patients with non-delaminated tears were available for postoperative study. According to the repair methods, 33 patients were treated with dual-layer double-row (DLDR) repair and 20 patients with dual-layer suture bridge (DLSB) repair technique. The 11 patients with non-delaminated tears underwent a single-layer repair. The mean age at the time of operation was 62.5 years (range, 44 to 79 years) in the dual-layer repair group and 63.7 years (range, 40 to 75 years) in the single-layer repair group. The mean follow-up periods after surgery were 26.2 months (range, 18 to 40 months) and 28.1 months (range, 19 to 38 months), respectively. According to the classification of DeOrio and Cofield [11], the extent of the tear was determined intraoperatively under direct arthroscopic visualization after debridement of the degenerated tendon edges. The tear size was measured in the anterior-posterior dimension using an arthroscopic probe. There were seven small, 33 medium and 13 large tears in the dual-layer repair group and one small, seven medium and three large tears in the dual-layer repair group (Table 1). The retraction direction of the deep and superficial layers was analyzed with arthroscopic findings and operative notes that described the reduction pattern of the delaminated rotator cuff.

**Table 1:** Patient demographics

Variables	Dual-layer repair group (*n* = 53)	Single-layer repair group (*n* = 11)	*p* value
Gender, male/female	25/18	4/7	0.522
Dominant/non-dominant	25/18	6/5	0.919
Tear size, small/medium/large	7/33/13	1/7/3	0.744
Mean age, year (SD)	62.5(7.6)	63.7(8.4)	0.859
Mean follow-up period, months (SD)	26.2(9.7)	28.1(10.5)	0.776
Anchors, no. (SD)	2.27(0.8)	3.84(0.46)	<0.001

MRI magnetic resonance imaging

Tendon integrity was evaluated by postoperative MRI performed at an average of 12.1 months after the surgery (range, 9 to 14 months). Twenty-six dual-layer rotator cuff repairs (14 DLDR, 12 DLSB) and 11 single-layer repairs were enrolled in this study. MRI was performed with the use of a 1.5-Tesla scanner (Magneton Essenza 1.5 T, Siemens, Germany). Postoperative cuff integrity was classified into five categories using Sugaya’s classification [9] for MRI.

### Tear pattern of delaminated rotator cuff tears (Himchan classification)

Randomized arthroscopic pictures were reviewed by two independent blinded observers, and patterns of delamination were assessed. The presence of delamination was evaluated at the time of arthroscopic operation, and the tear pattern was analyzed in accordance with the mobility and reduction pattern of each of the superficial and deep layers. The retraction direction of the deep layer was categorized into posteromedial (type D1) (Fig. 1) and anteromedial retraction (type D2) (Fig. 2). Superficial layers were classified into three categories as follows: (1) posteromedial retraction (type S1) (Fig. 3), majority of infraspinatus (ISP) involvement (ISP > SSP); (2) anteromedial retraction (type S2) (Fig. 4), majority of supraspinatus (SSP) involvement (SSP > ISP); and (3) bilateral retraction (type S3) (Fig. 5).

**Fig. 1:**
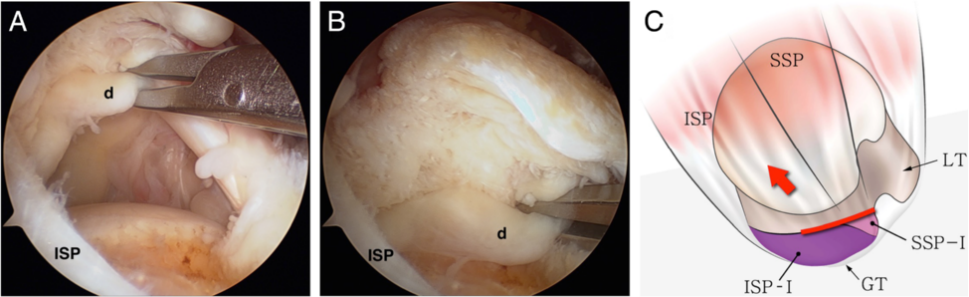
**a** Intraoperative arthroscopy shows posteromedial retraction of the deep layer (*d*) in a delaminated rotator cuff tear. **b** The retracted deep layer was reduced anterolaterally with a tissue grasper. **c** The illustration shows posteromedial retraction of the deep layer (type D1) in a delaminated rotator cuff tear. (*SSP* supraspinatus, *ISP* infraspinatus, *GT* greater tuberosity, *LT* lesser tuberosity, *ISP-I* footprint of infraspinatus [7], *SSP-I* footprint of supraspinatus [7]; *red line* deep layer or capsule [17])

**Fig. 2:**
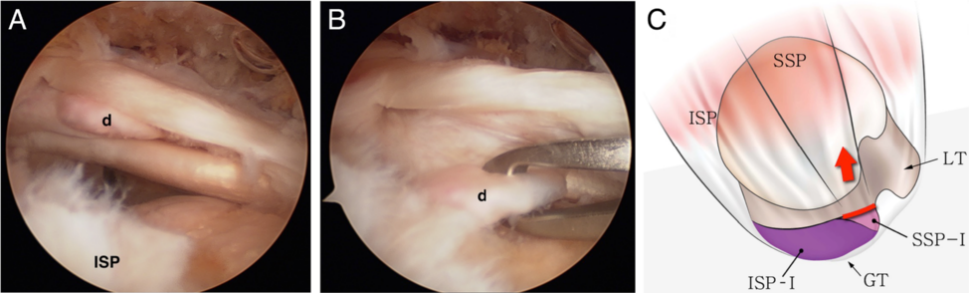
**a** Intraoperative arthroscopy shows anteromedial retraction of the deep layer (*d*) in a delaminated rotator cuff tear. **b** The retracted deep layer was reduced posterolaterally with a tissue grasper. **c** The illustration shows the anteromedial retraction of the deep layer (type D2) in a delaminated rotator cuff tear. (*SSP* supraspinatus, *ISP* infraspinatus, *GT* greater tuberosity, *LT* lesser tuberosity, *ISP-I* footprint of the infraspinatus [7], *SSP-I* footprint of the supraspinatus [7]; *red line* deep layer or capsule [17])

**Fig. 3:**
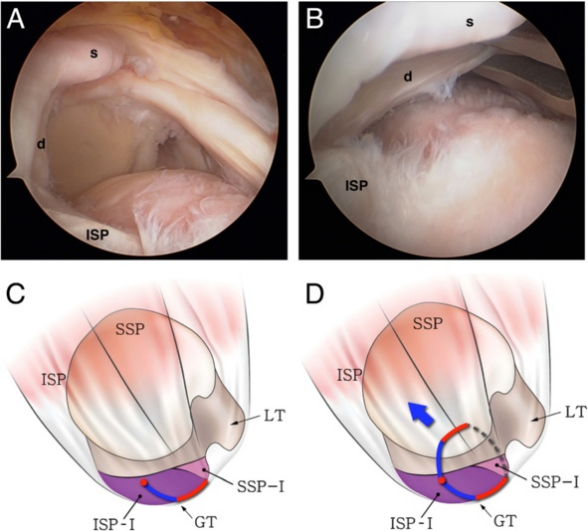
**a** Intraoperative arthroscopy shows posteromedial retraction of the superficial layer (*s*) in a delaminated rotator cuff tear. **b** The retracted superficial and deep (*d*) layers were reduced together by pulling the deep layer anterolaterally with a tissue grasper. **c** The illustration shows that the anterosuperior rotator cuff is detached from the greater tuberosity (*red line*) and the tear progressed into the cuff substance leading to longitudinal splitting through the infraspinatus (*blue line*). **d** A larger proportion of the infraspinauts (ISP > SSP) in the superficial layer causes posteromedial retraction (type S1) (*dotted line* elongated rotator interval tissue, *red circle* end of cuff tear, *SSP* supraspinatus, *ISP* infraspinatus, *GT* greater tuberosity, *LT* lesser tuberosity, *ISP-I* footprint of the infraspinatus [7], *SSP-I* footprint of the supraspinatus [7])

**Fig. 4:**
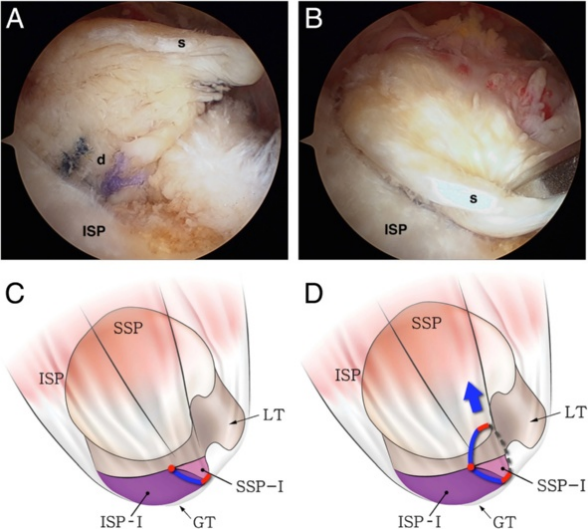
**a** Intraoperative arthroscopy shows the anteromedial retraction of the superficial layer (*s*) in a delaminated rotator cuff tear (the same case as in Fig. 1). **b** The retracted superficial layer was reduced posterolaterally with a tissue grasper. **c** The illustration shows that the anterosuperior rotator cuff is detached from the greater tuberosity (*red line*) and the tear progressed into the cuff substance, leading to longitudinal splitting through the supraspinatus or infraspinatus (*blue line*). **d** A larger proportion of the supraspinatus (SSP > ISP) in the superficial layer causes anteromedial retraction (type S2) (*dotted line* elongated rotator interval tissue, *red circle* end of cuff tear, *SSP* supraspinatus, *ISP* infraspinatus, *GT* greater tuberosity, *LT* lesser tuberosity, *ISP-I* footprint of the infraspinatus [7], *SSP-I* footprint of the supraspinatus [7])

**Fig. 5:**
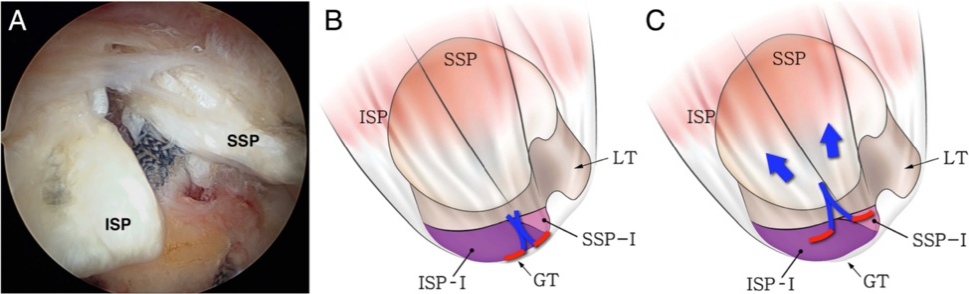
**a** Intraoperative arthroscopy shows bilateral retraction of the superficial layer in a delaminated rotator cuff tear. **b** The illustration shows that the anterosuperior rotator cuff is detached from the greater tuberosity (*red line*) and the tear progressed into the cuff substance, leading to longitudinal splitting between the supraspinatus and infraspinatus (*blue line*). **c** The supraspinatus and infraspinatus in the superficial layer are retracted bilaterally (type S3) (*SSP* supraspinatus, *ISP* infraspinatus, *red circle* end of cuff tear, *GT* greater tuberosity, *LT* lesser tuberosity, *ISP-I* footprint of the infraspinatus [7], *SSP-I* footprint of the supraspinatus [7])

### Clinical assessment

Clinical data were collected both on the day prior to surgery and postoperatively at 3, 6, 12, and 24 months. Five outcome measures were used in this study: VAS pain score, UCLA score, Constant score, active shoulder range of motion (ROM), and rotator cuff muscle strength.

### Operative techniques

All procedures were performed by the senior author (CK Lee) with the patient in the beach-chair position and the back of the bed flexed approximately 70°. A posterior portal was established for the initial assessment of the glenohumeral joint and the deep layer of the delaminated rotator cuff tear. An anterior portal through the rotator interval was established as the working portal for the treatment of intraarticular lesions. The arthroscope was then removed from the glenohumeral joint and redirected into the subacromial space. An anterolateral portal and posterolateral portal were also established. Acromioplasty was performed in all cases. The tear size and pattern were evaluated through the posterolateral portal as the main viewing portal. The tear retraction pattern was evaluated using a KingFisher retriever/grasper (Arthrex, Naples, FL) with the direction of the reduction of the deep and superficial layers (Figs. 1, 2, 3, 4, and 5), and in the case of insufficient mobility of the rupture cuff, capsule tissues in the lower portion of the deep layer around the scapular spine and coracoid process were released. Decortication on the greater tuberosity was performed using a burr to enhance cuff healing.

DLDR rotator cuff repair [8] repairs the deep and superficial layer separately based on the direction of the tear retraction of the two layers. One or two anchors were inserted at 5 mm lateral to the articular margin in order to obtain enough space for the delaminated deep layer. In the case of the posteromedial retraction of the deep layer, sutures passing through the deep layer were executed using a suture lasso (Arthrex, Naples, FL) while reducing it with the KingFisher anterolaterally. It is important to confirm that the tip of the lasso protrudes out of the articular surface of the deep layer. The sutures were tied with a sliding SMC knot [12]. Anatomical deep layer repair causes the superficial layer to reduce to a certain extent. Anatomical superficial layer repair is performed through the same method as deep layer repair. After inserting one to three anchors into the lateral margin of the greater tubercle, the superficial layer suturing was performed while reducing the superficial layer either anterolaterally or posterolaterally depending on the retraction pattern (Fig. 6a, b).

**Fig. 6:**
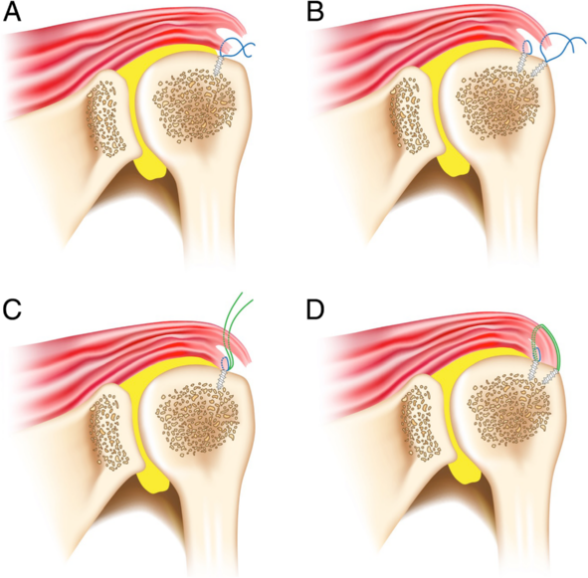
**a**, **b** Dual-layer double row (DLDR) rotator cuff tear. **c**, **d** Dual-layer suture bridge (DLSB) rotator cuff tear

There is also a second dual-layer repair method. If the size of the tear in the deep layer is wide enough to insert two anchors into the medial row, DLSB rotator cuff repair can be performed. After performing anatomical deep layer repair with only one or two strands of each anchor in the same manner as DLDR repair, suture bridge repair of the superficial layer was performed using Versalok (DuPey Synthes, Raynham, MA) by passing the one remaining suture thread or one of the two tied threads from each anchor in a reduced state (Fig. 6c, d). DLSB technique is thought to be an effective surgical method if the tear retraction of the deep layer and the superficial layer are in the same direction. The repaired deep layer was observed from the posterior portal after DLDR or DLSB rotator cuff repair.

### Postoperative rehabilitation

An abduction brace was administered for 4 to 6 weeks after the surgery in accordance with the size of the tear. Isometric rotator cuff exercises and relaxation of the muscles around the shoulder were initiated immediately following the surgery. Passive ROM exercise began from the second week after the surgery, and active assisted ROM exercise began on the eighth. Forward flexion and external rotation exercises were conducted within pain-free ROM and active ROM, and muscle strengthening exercise began on the 12th week following the surgery. Three months after the surgery, the intensity of exercise was increased gradually, and a full return to athletic activity and/or heavy labor were allowed on the sixth month following the surgery.

### Statistical analysis

Patient samples were determined by repair type (dual layer vs. single layer). Preoperative and postoperative non-parametric data from both groups were analyzed with a Wilcoxon-signed rank test. Comparison between the two groups was performed by use of the Mann-Whitney *U* test. The *k* value for interobserver reliability was measured. Significance was set at as *α* level of 0.05 with 95 % confidence intervals (GraphPad Prism version 5.0 for Macintosh, GraphPad Software Inc., San Diego, CA).

## Results

### Delamination

The incidence of delamination was 81.3 % or 53 cases among the 64 patients. The rate of posteromedial (47 cases), anteromedial (five cases), and bilateral (one case) retraction in the superficial layer was 88.7, 9.4, and 1.9 %, respectively (Figs. 3, 4, and 5). All patients excepting the single case (98.1 %) had posteromedial retraction in the deep layer of delaminated rotator cuff tear (Figs. 1 and 2). The *k* value for interobserver reliability in detection of tear pattern was 0.817.

### Clinical outcomes

The UCLA score and Constant score reflected a significant improvement in the status of the shoulders when the preoperative scores were compared with those at the final follow-up (*p* < 0.001). The Constant score of the dual-layer repair group increased from the preoperative mean of 58.4 ± 7.6 points to 75.8 ± 10.9 points at the final follow-up (*p* < 0.001). The corresponding figures for the single-layer group improved from 57.7 ± 4.2 points to 78.4 ± 10.9 points (*p* < 0.001). The preoperative UCLA score was 16.8 ± 2.5 points in the dual-layer repair group and 17.3 ± 2.2 points in the single-layer repair group. The UCLA score at the final follow-up was 29.6 ± 4.6 points in the dual-layer repair group and 30.0 ± 4.5 points in the single-layer repair group (Table 2). However, there was no statistical difference between the groups in the average postoperative total scores with use of the Constant score (*p* = 0.0347) or the UCLA score (*p* = 0.297) (Table 2).

**Table 2:** Comparison of clinical outcomes between the dual-layer repair and single-layer repair groups

Variable	Dual-layer repair group (*n* = 53) Mean (SD)	Single-layer repair group (*n* = 11) Mean (SD)	Dual-layer repair vs. single-layer repair *p* value
Preoperative			
VAS (scale 1–10)			
FF	128.2 (48)	130.4 (55)	0.532
ERs	41.9 (39)	35.7 (30)	0.135
IRP	L3.5	L4.0	0.426
UCLA score	16.8 (2.5)	17.3 (2.2)	0.773
Constant score	58.4 (7.6)	57.7 (4.2)	0.542
Follow-up			
VAS (scale 1–10)	1.7 (1.1)	2.2 (1.7)	0.145
FF	161.9 (43)	160.7 (52)	0.702
ERs	51.9 (36)	55.7 (31)	0.333
IRP	T10.5	T10.7	0.321
UCLA score	30.0 (4.5)	29.6 (4.6)	0.347
Constant score	75.8 (10.9)	78.4 (10.9)	0.297

*VAS* visual analog scale, *ROM* range of motion, *FF* forward flexion, *ERs* external rotation at the side, *IRP* internal rotation to the posterior, *ER* external rotation, *IR* internal rotation, *UCLA* the University of California at Los Angeles

### Structural outcomes

The follow-up MRI in the 26 cases in the dual-layer repair group revealed 12 type I, six type II, six type III, and two type V results according to Sugaya’s classification. There were two re-tears (7.6 %), one in the DLDR group and the other in the DLSB group. The follow-up MRI in the 11 cases of single-row repair revealed five type I, three type II, and three (27.2 %) type V results (Table 3). A statistical difference was observed between the two groups (*p* = 0.016). Three of the six cases that demonstrated Sugaya type III results were observed in the DLDR group, while the other three were observed in the DLSB group (Fig. 7). Among the 18 with Sugaya types I and II structural integrity, two cases showed sustained delamination under MRI following surgery (11 %), but the delamination disappeared in 16 cases (89 %).

**Table 3:** Structural integrity of arthroscopic dual-layer rotator cuff repair

Sugaya’s cuff grade	I	II	III	IV	V	Total
Single row (SR)	5	3			3	11
Dual-layer double row (DLDR)	7	3	3	0	1	14
Dual-layer suture bridge (DLSB)	5	3	3	0	1	12
Total	17	9	6	0	5	37

**Fig. 7:**
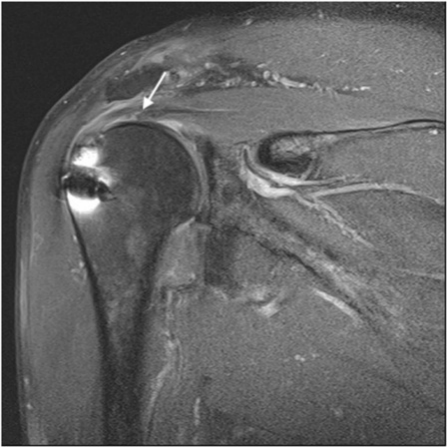
A postoperative MRI of a Sugaya type III result shows deep layer re-tear (white arrow)

## Discussion

The purpose of this study was to determine the incidence of delaminated cuff tears and analyze their retraction patterns. Dual-layer (anatomically balanced) rotator cuff repair based on the retraction patterns of delaminated rotator cuff tears may achieve a normal footprint for the rotator cuff on the greater tuberosity and optimize the healing rate. By analyzing the progression direction of both the superficial and deep layers of delaminated cuff tears, we found that delaminated cuff tears mainly retracted in a posteromedial direction based on the anatomic features of the supraspinatus and infraspinatus footprints and their fiber directions, an observation not made in prior studies.

According to a recent anatomic study [7], the insertion area of the supraspinatus into the highest impression of the greater tuberosity is much smaller than has been previously reported, and this area of the greater tuberosity is actually occupied to a substantial degree by a portion of the infraspinatus. Several clinical studies have reported that muscle atrophy in the infraspinatus, frequently observed in isolated supraspinatus tears, may be due to the retraction of the suprascapular nerve in shoulders with rotator cuff tears [13–15]. However, Mochizuki et al. [7] suggested that infraspinatus involvement in rotator cuff tears occurred with high frequency, contributing to infraspinatus muscle atrophy and weakness in external rotation and shoulder abduction. Such findings support the concept that although a tear may be localized to the highest impression or small in size, the superficial layer of delamination may include not only the supraspinatus but also various portions of the infraspinatus depending on the size of an anteroposterior cuff tear.

Mochizuki et al. [7] stated that the infraspinatus is divided into long, thick fibers and short, thin fibers, each running in different directions, and a long tendinous portion in the superior half of the muscle is curved anteriorly and extends to the anterolateral area of the highest impression of the greater tuberosity. Such anatomic features of the infraspinatus may have an impact on the progression direction of the superficial layer of delaminated rotator cuff tears. In the present study, three tear retraction patterns—running in posteromedial (type S1), anteromedial (type S2), and bilateral (type S3) directions—were observed. Among the three patterns, the incidence of the posteromedial direction was highest at 88.7 % (Figs. 3, 4, and 5).

These patterns may be attributed to several factors. Kim et al. [16] previously showed that degenerative rotator cuff tears were most commonly initiated from a region 13–17 mm posterior to the biceps tendon. Nimura et al. [17] noted that 11 mm posterior to the anterior margin of the greater tuberosity was the thinnest point of the articular capsule and very close to the posterior edge of the tapered insertion of the supraspinatus, which may lead to degenerative rotator cuff tears. From this point, rotator cuff tears are expected to run anteriorly, posteriorly, or bilaterally, and the retraction pattern of the superficial layer may be determined by the amount of the infraspinatus tendon involved in the tear. In the case of posteromedial retraction (type S1), the portion of the infraspinatus in the superficial layer is larger than that of the supraspinatus, allowing tears to retract in the infraspinatus fiber direction. On the other hand, in the case of anteromedial retraction (type S2), the superficial layer contains a larger amount of the supraspinatus, leading the retraction in the supraspinatus fiber direction. The remaining tendinous portion of the posterior rotator cuff is frequently observed at the lateral end of the greater tuberosity, which is easily mistaken for the remaining intratendinous tear and completely removed for the sake of tendon-to-bone healing. The remaining tendinous portion, however, may be part of a longitudinal split of the infraspinatus (Figs. 3a and 4a) extending from the cuff tear. Since connection to the infraspinatus tendon is the normal tendon structure, care should be taken not to remove it. The retracted cuff tendon may also contain part of a longitudinal split of the infraspinatus. It is of essence to reduce the longitudinal split of the infraspinatus for anatomic rotator cuff repair (Figs. 3 and 4).

Nimura et al. [17] conducted an anatomic study on the attachment of the articular capsule on the greater tuberosity by removing the rotator cuff. The width of the articular capsule was measured between 3.5 and 9.1 mm, with a thicker footprint than previously believed. Their study suggests that a large portion of the deep layer observed in delaminated rotator cuff tears may be composed of the articular capsule. However, given the finding that the deep layer reached the musculotendinous junction on the MRI scans of patients with delaminated rotator cuff tears, the deep layer is considered a structure that serves as a tendon connected to the infraspinatus and supraspinatus rather than a static structure consisting of the capsule alone. In this regard, the retraction pattern of the delaminated deep layer may be affected by the infraspinatus and supraspinatus. In our series, 98.1 % of the delaminated deep layer progressed in the posteromedial direction, leading to the assumption that the deep layer is most likely associated with the infraspinatus and works as a tendon. Some authors have reported that the presence of delamination to be a negative prognostic factor of the anatomic results in rotator cuff repair [1, 2]. However, none of them discussed a specific treatment method for delamination. Zilber et al. [10] noted that delamination of the infraspinatus did not affect the outcome of supraspinatus repair. However, eight (28 %) of their 29 patients showed grade II fatty infiltration of the infraspinatus muscles, despite them not being torn. In addition, weakness in external rotation was found in half of their patients. They also reported that nine supraspinatus tendons (31 %) were continuous but thin. These findings imply that their treatment option for the infraspinatus delamination may have been improper, and re-tear of the deep layer may have caused fatty infiltration, weakness in the infraspinatus, and thinning of the repaired supraspinatus tendon. In the present study, we also observed thinning of the repaired rotator cuff (Sugaya classification III) caused by a re-tear of the deep layer in six (24 %) of the 26 cases (Fig. 7). Thinning of the repaired cuff would deteriorate with a deep layer re-tear, leading to elongation of the sutured superficial layer and a complete re-tear of the cuff. Repair of the superficial layer alone without addressing the deep layer may result in thinning of the rotator cuff as well as weakness in abduction and external rotation and have a negative impact on the results after cuff repair.

Sugaya et al. [8] emphasized the importance of precise evaluation of delamination and cuff mobilization for good clinical results and recommended DLDR repair for improved structural integrity of the delaminated rotator cuff. In the present study, we assumed that dual-layer repair (anatomically balanced repair) of delaminated rotator cuff tears would achieve optimal results by restoring each layer to its original anatomic state. In most cases, the deep layer may be easily reduced to its original position when pulled anterolaterally since the deep layer is found retracted in the posteromedial direction (type D1). Both DLSB and DLDR repairs may be performed when a tear of the deep layer is wide enough to fix two anchors for repair, the retraction directions of respective layers are identical, and the superficial layer matches the anatomic footprint relatively well. In our series, a complete re-tear (Sugaya type V) was observed in two (8 %) of the 28 cases. There were six cases of Sugaya type III thinning, all of which involved re-tearing of the deep layer. When degeneration of the deep layer is severe, conventional single-layer double-row repair is more desirable than dual-layer repair because a deep layer re-tear caused by degeneration could eventually lead to the failure of the repaired rotator cuff.

Sonnabend et al. [6] recommended layer-to-layer repair with curettage of the synovial lining, since the presence of the synovial covering induced lamination and interfered with the healing of the layers to each other. In the present study, however, delamination remained in only two out of 28 patients following anatomically balanced (dual-layer) repair. Accordingly, the patients recovered from delamination without curettage.

Our study features certain limitations. First, being retrospective in nature, it includes a relatively small number of enrolled patients, a limitation shared with other retrospective studies. Second, the follow-up period is relatively short compared with other long-term studies on rotator cuff repair. Third, with regard to preoperative or postoperative imaging evaluation, analysis of postoperative repair integrity using MRI was not conducted in all cases. Fourth, the presence of delamination and the retraction pattern of the superficial and deep layers were somewhat subjective. We attempted to overcome this limitation by involving multiple surgeons and independent observers. Lastly, our strategies for the treatment and repair of delaminated lesions were those that we applied in practice, but they have not been validated as superior by direct comparative studies. Despite these limitations, our study presents the advantage of providing an analysis of delaminated tearing patterns and structural outcomes after repair, while previous studies simply compared functional results according to the presence of delaminated rotator cuff tears.

## Conclusion

Differential rotator cuff repair strategies are needed to address rotator cuff tears, since recent studies have changed our concept of rotator cuff tears. We focused on three subjects in regard to delamination: incidence, retraction patterns and clinical outcomes. The incidence of delamination was higher than expected at over 80 %. Both the superficial and deep layers in the delaminated cuff tears mainly retracted posteromedially. The retraction of the deep layer and superficial layer may be affected by the infraspinatus. Awareness of delamination and retraction patterns allows anatomical balancing of each layer of delamination and appropriate tensioning of the rotator cuff repair. Dual-layer rotator cuff repair based on the retraction pattern of delamination is recommended for improved repair integrity and better clinical outcomes.
